# Highly sensitive determination of lead(ii) and cadmium(ii) by a large surface area mesoporous alumina modified carbon paste electrode

**DOI:** 10.1039/c8ra00041g

**Published:** 2018-02-19

**Authors:** Xinyu Zheng, Shen Chen, Jiebo Chen, Yuheng Guo, Jun Peng, Xuechou Zhou, Rixin Lv, Jiandi Lin, Ruiyu Lin

**Affiliations:** School of Life Sciences, Fujian Agriculture and Forestry University Fuzhou Fujian 350002 China mailto:zhengxinyu0621@sina.com zhengxinyu0621@sina.com ruiyulin121011@sina.com; College of Chemistry, Fuzhou University Fuzhou Fujian 350116 China

## Abstract

Nanosized mesoporous γ-alumina (M-γ-Al_2_O_3_) was first prepared and then modified into a carbon paste to fabricate a novel modified carbon paste electrode. The prepared alumina has pores with an amorphous wall and large surface area. The electrochemical behavior of the modified carbon paste electrode was investigated using cyclic voltammetry (CV) and electrochemical impedance spectroscopy (EIS) methods. The modified carbon paste electrode was employed to determine Pb^2+^ and Cd^2+^ simultaneously by a differential pulse voltammetry (DPV) method. Amperometric determination was carried out in 0.1 mol L^−1^ NaAc–HAc buffer solution (pH 6.0) after enriching for 360 s at −1.0 V. The oxidation peak currents of Pb^2+^ and Cd^2+^ were proportional to their concentration in the range of 0.001–10 μmol L^−1^ and 0.01–10 μmol L^−1^, respectively. The detection limits of Pb^2+^ and Cd^2+^ were 0.20 nmol L^−1^ and 2.0 nmol L^−1^ (S/N = 3), respectively. The modified carbon paste electrode shows good stability, repeatability and sensitivity. The proposed method was applied to the determination of Pb^2+^ and Cd^2+^ in water samples with satisfactory results.

## Abstract 2

Nanosized mesoporous γ-alumina (M-γ-Al_2_O_3_) was first prepared and then modified into a carbon paste to fabricate a novel modified carbon paste electrode.

## Graphical abstract

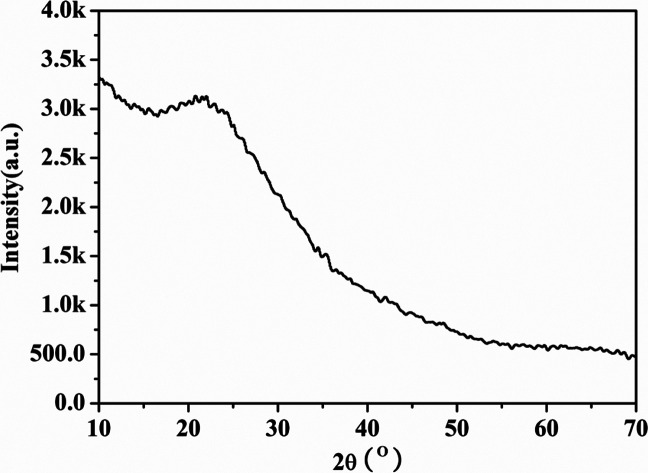

## 1. Introduction

Lead and cadmium are both toxic heavy metal elements. Because of their toxicity and non-biodegradability, they can not only be accumulated in the body and endanger human health, but also cause serious pollution to the environment.^1–3^ Consequently, knowledge of the quantities of Pb^2+^ and Cd^2+^ in environmental samples has important practical significance. At present, various analytical techniques have been reported for the determination of Pb^2+^ and Cd^2+^ at low concentrations, such as atomic fluorescence spectrometry,^4,5^ atomic absorption spectroscopy,^6,7^ inductively coupled plasma mass spectrometry,^8,9^ and high performance liquid chromatography.^10^ Although these techniques have good selectivity and high sensitivity, most of the above techniques require relatively ponderous and complicated instruments and are time-consuming, in particular for the *in situ* analysis of metal ions. In contrast, electrochemical voltammetry is widely used for trace heavy metal ion analysis and on-site testing because of its low-cost instruments and equipment, high sensitivity, wide detection range, rapid performance and high portability.

The carbon paste electrodes have the advantages of simple preparation, non-toxicity, chemical inertness, very stable electrochemical response, easy updating the surface, long operational lifetime and low background current.^11,12^ Owing to these advantages, carbon paste electrodes have become one of the most popular electrode materials. The selectivity and sensitivity of the carbon paste electrodes may be improved by adding special modifiers to the carbon paste.^13^ However, it is still a challenging requirement to design or synthesize a new type of modifier for higher selectivity and sensitivity.

Mesoporous materials, such as Ni-ZSM-5, Ni-SBA-15, Cu-zeolite and Pb-zeolite, were widely applied in catalysts, sensors and energy storage due to their high specific surface area, large pore volume, narrow pore size distribution, tunable pore size and favorable stability.^14–17^ Research indicates that M-γ-Al_2_O_3_ can raise the electrode conductivity, the electron transfer and can also enhance the analytical sensitivity due to their large surface areas. Up to now, many electrochemical sensors have been reported based on M-γ-Al_2_O_3_ with ordinary structure for the determination of sodium aerosol, nicotinamide adenine dinucleotide, amitriptyline, captopril, ascorbic acid, dopamine and uric acid, and so on.^18–20^

Many approaches have been used to synthesize large surface area mesoporous alumina with the P123 as a structure-directing agent.^21,22^ The high surface area alumina are prepared *via* mainly controlled the Al^3+^ hydrolysis speed by regulating pH value and reducing the concentrations of anions in the reaction system to disturb the self-assembly process.^23–25^ In process of the synthesis, the added aniline could not only regulate the pH value of the reaction system to control the Al^3+^ hydrolysis speed, but also could protect the aluminum ions at the organic–inorganic interface from being affected by the chloride ions of the reaction system. So in present work, aniline was chosen as an interface protective agent to prepare large surface area mesoporous alumina which was employed to modify the carbon paste. The as-prepared large surface area mesoporous alumina modified carbon paste was used to prepare modified carbon paste electrode. The prepared M-γ-Al_2_O_3_ was characterized by powder X-ray diffraction (PXRD), transmission electron microscopy (TEM) and Brunauer–Emmett–Teller (BET) analysis. Cyclic voltammetry (CV) and electrochemical impedance spectroscopy (EIS) methods were utilized to study the electrochemical behavior of the novel working electrode. A DPV method was developed for the simultaneous determination of Pb^2+^ and Cd^2+^. The results show that the modified carbon paste electrode has excellent sensitivity, wide linear range, low detection limit, favorable stability and reproducibility. Finally, the modified carbon paste electrode was successfully applied to the determination of Pb^2+^ and Cd^2+^ in water samples.

## 2. Experimental

### 2.1. Apparatus

The CHI660D Electrochemical Workstation (Shanghai Chenhua Instruments, Shanghai, China) was used to perform the electrochemical measurements. The three electrodes electrochemical cell contain an unmodified or modified carbon paste electrode with a diameter of 3.0 mm and 5.0–6.0 mm in depth (working electrode), a platinum electrode (auxiliary electrode) and Ag/AgCl/sat. KCl electrode (reference electrode). Powder X-ray diffraction (PXRD) analysis was conducted by a PANalytical X'pert diffractometer with Cu Kα (1.5418 Å) radiation generated at 40 kV and 40 mA. The surface area (calculated by BET method), pore size distribution (calculated by BJH method) and pore volume were obtained at −196 °C using an Ominsorp 100cx analyzer (Micromeritics Tristar, USA). Transmission electron microscopy (TEM) images were acquired by a JEM 2100 electron microscope operating at an accelerating voltage of 200 kV.

### 2.2. Chemicals and materials

Pb(NO_3_)_2_, Cd(NO_3_)_2_·4H_2_O, Al_2_O_3_, graphite powder, liquid paraffin, aniline and aluminum isopropoxide were bought from Sinopharm Chemical Reagent Co., Ltd. Pluronic P123 (Mav = 5800, EO_20_PO_70_EO_20_) was obtained from Aldrich Chemical Company (Shanghai, China). Other reagents were analytical grades and purchased from local commercial sources. Buffer solution was prepared by mixing 0.1 mol L^−1^ NaAc and 0.1 mol L^−1^ HAc solutions to form a 0.1 mol L^−1^ NaAc–HAc buffer solution. Distilled water was used for the preparation of all solutions and for washing. Both standard solution and buffer solutions were kept in a 4 °C refrigerator.

### 2.3. Preparation of large surface area M-γ-Al_2_O_3_

In order to prepare the large surface area M-γ-Al_2_O_3_, a delicate procedure should be designed. Firstly, 1 g of Pluronic P123 was dissolved in 20 mL of the absolute ethanol to form a solution. Then quantitative aluminum isopropoxide, HCl and aniline were added successively to the solution. This mixture was stirred at room temperature until the entire solid was dissolved completely. Consequently, a homogeneous sol was obtained. Secondly, the homogeneous sol was transferred to an oven. The solvent was evaporated with a temperature at 40 °C for 48 h to prepare a gel product. After that, the gel product was dried at 100 °C for 24 h. Finally, the as-prepared sample was calcined at 400 °C in a muffle furnace with a heating rate of 10 °C min^−1^ and held for 3 h at the maximum calcination temperature. The large surface area M-γ-Al_2_O_3_ was synthesized successfully after the above steps.

### 2.4. Preparation of modified carbon paste electrode

The modified carbon paste electrode was prepared as following process. M-γ-Al_2_O_3_, graphite powder and liquid paraffin were mixed together in definite proportions. The mixture was grinded for about 10 minutes to get a homogenous carbon paste. Small amount of the obtained carbon paste was taken to pack tightly into the tip cavity of a carbon paste electrode. This electrode is defined as M-γ-Al_2_O_3_-CPE. For comparison purpose, bare carbon paste electrode (BCPE) can be manufactured by the similar way without adding M-γ-Al_2_O_3_. Smooth surfaces of M-γ-Al_2_O_3_-CPE and BCPE can be obtained by polishing the electrodes on a weighting paper.

### 2.5. Electrochemical measurements

DPV method was carried out in a 10 mL volume of electrochemical cell containing 0.1 mol L^−1^ NaAc–HAc buffer solution (pH 6.0) as supporting electrolyte medium. The accumulation potential of Pb^2+^ and Cd^2+^ is −1.0 V with an accumulation time of 360 s. The optimum conditions of DPV method were determined. The potential ranges from −1.2 to 0 V. The pulse amplitude, the pulse width and the scan rate are 50 mV, 50 ms and 100 mV s^−1^, respectively. The calibration curves were obtained by plotting the peak current *versus* the Pb^2+^ and Cd^2+^ concentration. All electroanalytical measurements were conducted at room temperature.

## 3. Results and discussion

### 3.1. Characterization of M-γ-Al_2_O_3_

**Fig. 1:**
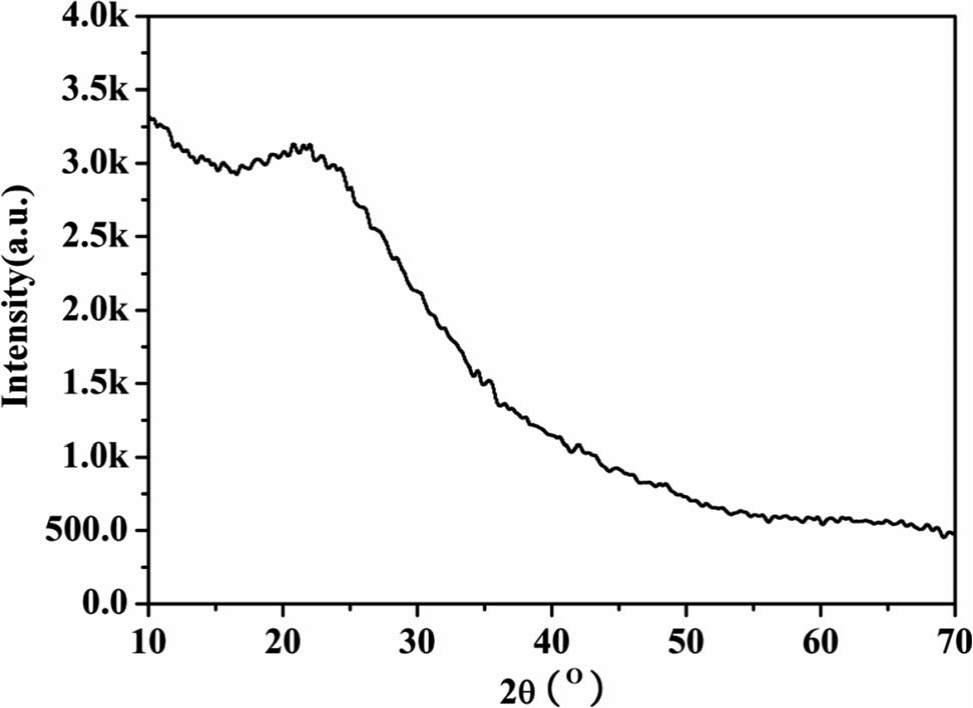
The PXRD spectrum of the large surface area M-γ-Al_2_O_3_.

**Fig. 2:**
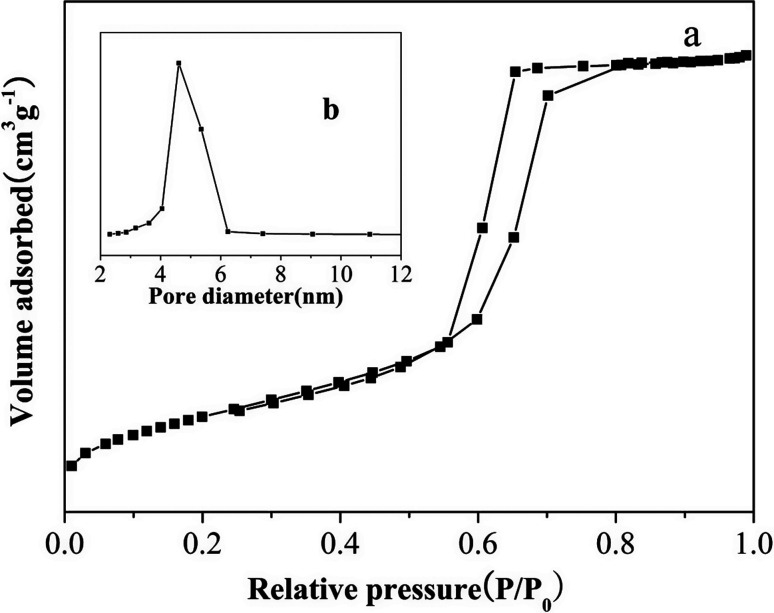
(a) Nitrogen adsorption–desorption isotherm and (b) pore-size distribution of the large surface area M-γ-Al_2_O_3_.

**Fig. 3:**
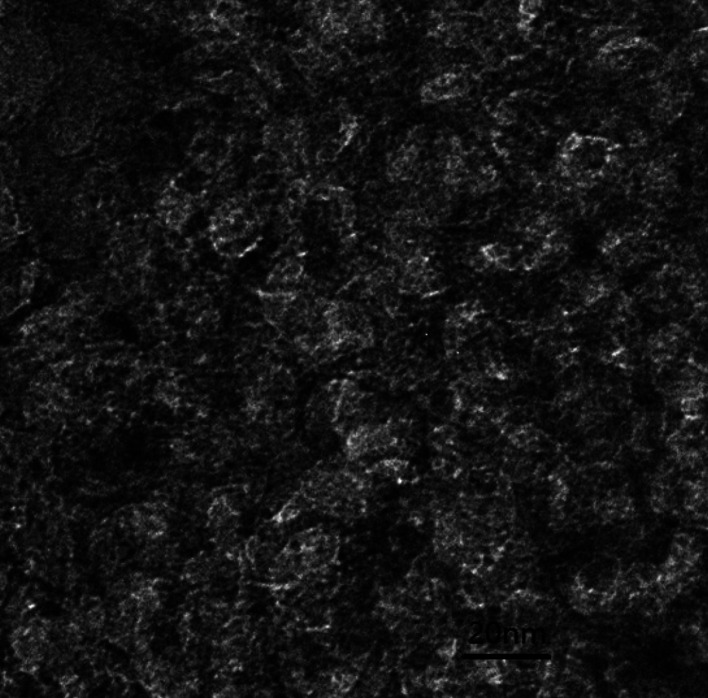
The TEM image of the large surface area M-γ-Al_2_O_3_.

The PXRD pattern of large surface area M-γ-Al_2_O_3_ is shown in Fig. 1. Only one flat peak can be found at 24°, which demonstrates the crystallinity of M-γ-Al_2_O_3_ is low and the M-γ-Al_2_O_3_ has pores with amorphous wall according to Yang *et al.*^26^Fig. 2 shows the nitrogen adsorption–desorption isotherms and pore size distribution curve of the M-γ-Al_2_O_3_. The synthetic M-γ-Al_2_O_3_ shows type IV curve with hysteresis loop at high pressure, which implies the existence of mesoporous pores. The isotherms exhibit distinct capillary condensation steps, indicating uniform mesoporosity. The synthetic M-γ-Al_2_O_3_ possesses a large surface area of 522 m^2^ g^−1^, a pore volume of 0.38 cm^3^ g^−1^, and a narrow pore-size distribution centered at 4.9 nm. The TEM image of large surface area M-γ-Al_2_O_3_ is shown in Fig. 3. The image reveals that the sample has lath-like appearance without significant order of pore arrangement. The grain diameters of sample are in the range of 10–20 nm. Hence, the very weak intensity of these diffraction peaks in the PXRD patterns for the sample could be attributed to their nanosized structure.

The improved surface area and comparable narrow pore size distribution were obtained compared with the prior literatures^21,27,28^ which used the P123 as a structure-directing agent for synthesizing the M-γ-Al_2_O_3_. In the synthesis system of mesoporous alumina synthesized by sol–gel method, the Cl^−^ from the acidity adjustment will be coordinated with Al^3+^, and the charge balance between the organic template and inorganic aluminum will be destroyed, thus interfere the self-assembly process. The addition of aniline can coordinate with Al^3+^ and inhibit the coordination between them, thus promoting mesoporous structure formation. Therefore, it is considered that aniline is an interfacial protective agent for forming mesoporous alumina.^29–31^ So aniline was chosen as interface protective agent to prepare the mesoporous alumina with large surface area. The synthetic M-γ-Al_2_O_3_ promise its potential application in the field of modified electrode.

### 3.2. The modified proportion of mesoporous alumina

**Fig. 4:**
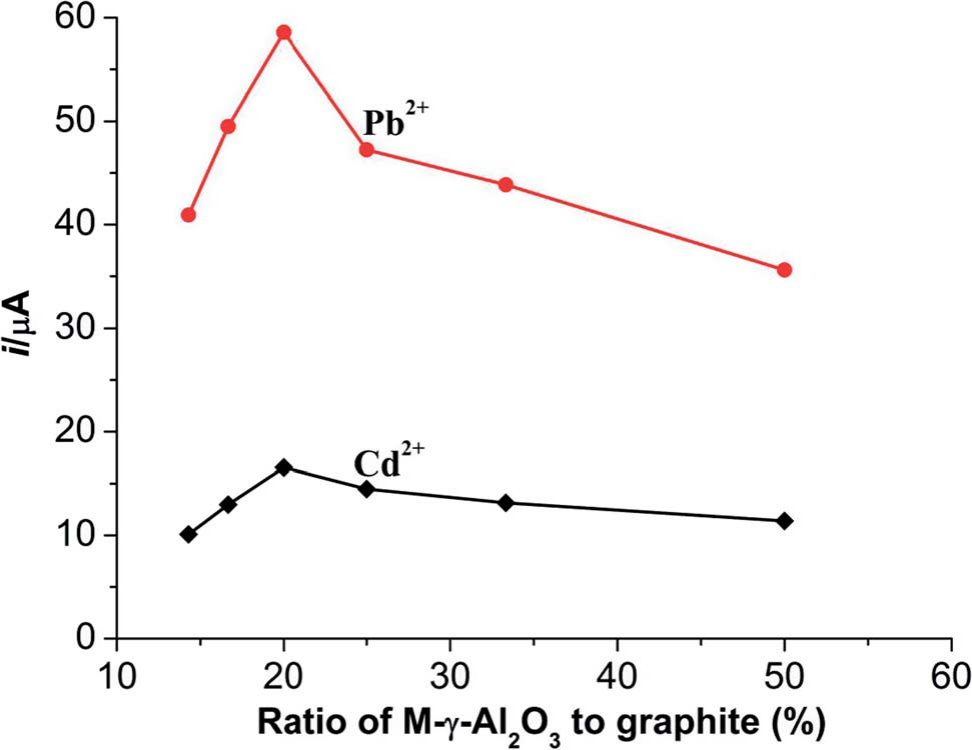
Influences of different mass ratios (M-γ-Al_2_O_3_ to graphite powder) on the current responses of 10 μmol L^−1^ Cd^2+^ and 10 μmol L^−1^ Pb^2+^. Supporting electrolyte: 0.1 mol L^−1^ NaAc–HAc buffer solution (pH 4.5); accumulation potential: −1.0 V; accumulation time: 180 s; pulse amplitude: 0.05 V; pulse width: 0.05 s; pulse period: 0.2 s.

The mass ratio of the solid mixture (graphite powder and M-γ-Al_2_O_3_) to liquid paraffin was fixed as 3 : 1. Influences of different mass ratios (M-γ-Al_2_O_3_ to graphite powder) on the current responses of Pb^2+^ and Cd^2+^ were well investigated. The results are shown in Fig. 4. The current responses of Pb^2+^ and Cd^2+^ enhance gradually to a maximum value and then decrease with the increasing proportion of M-γ-Al_2_O_3_. As can be known in Fig. 4, the optimal mass ratio of M-γ-Al_2_O_3_ to graphite powder is 1 : 4. The probably reasons may be explained that the current responses of two metal ions can increase significantly with the increasing proportion of M-γ-Al_2_O_3_ due to the properties of large surface area of the synthetic M-γ-Al_2_O_3_.

### 3.3. The proportion of liquid paraffin

The modified proportion of liquid paraffin was adjusted gradually to study its effect on the current response of Pb^2+^ and Cd^2+^. The mass ratios of the solid mixture (graphite powder and M-γ-Al_2_O_3_) to liquid paraffin was set as 5 : 1, 4 : 1, 3 : 1, 2 : 1, 1 : 1, respectively. The smaller amount of the liquid paraffin is used, the higher current responses of Pb^2+^ and Cd^2+^ can be achieved. If the proportion of added liquid paraffin is too small, the carbon paste could fall into the electrolyte. Good current responses and peak shapes of two metal ions can be achieved when the mass ratios of the solid mixture (graphite powder and M-γ-Al_2_O_3_) to liquid paraffin is 3 : 1.

### 3.4. The electrochemical property of M-γ-Al_2_O_3_-CPE

**Fig. 5:**
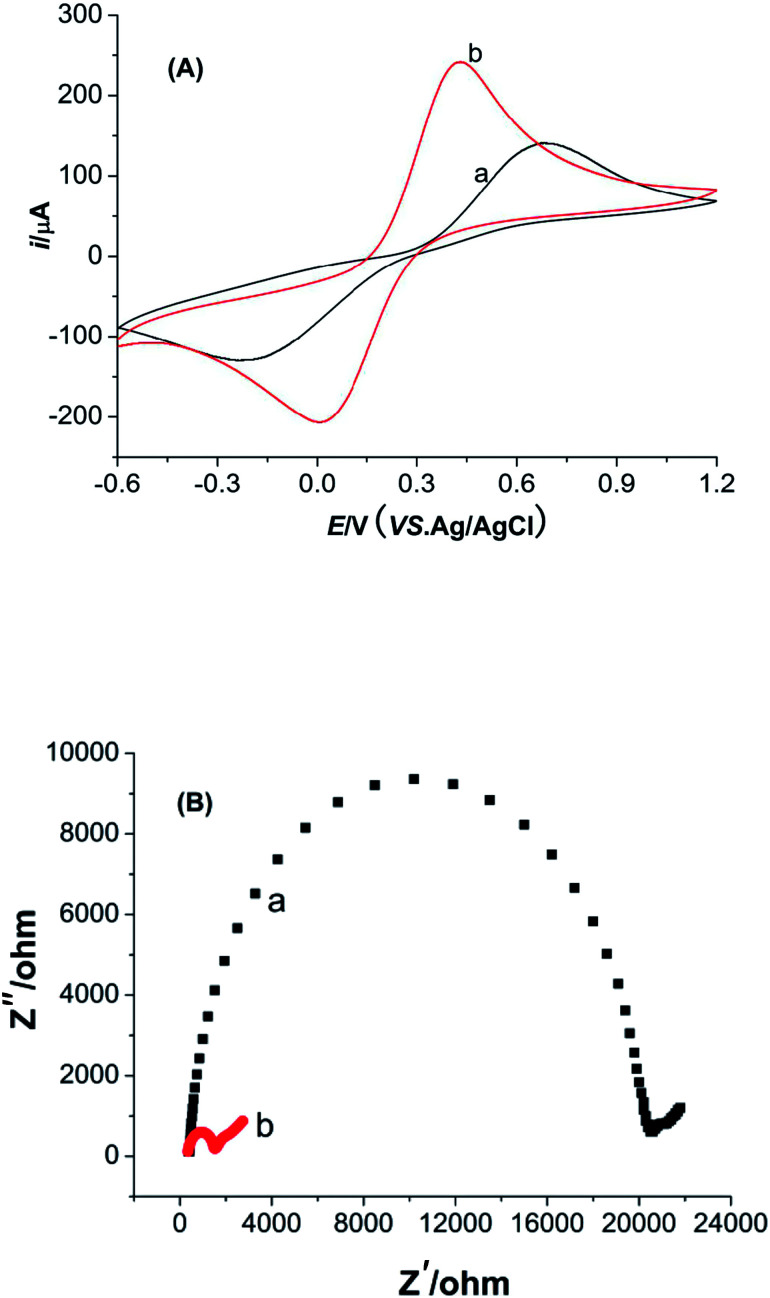
(A) CVs of BCPE (curve a) and M-γ-Al_2_O_3_-CPE (curve b) in 5.0 mmol L^−1^ K_4_[Fe(CN)_6_]/K_3_[Fe(CN)_6_] (1 : 1) solution containing 0.1 mol L^−1^ KCl. Scan rate: 100 mV s^−1^. (B) Nyquist plots of EIS in 5.0 mmol L^−1^ K_4_[Fe(CN)_6_]/K_3_[Fe(CN)_6_] (1 : 1) solution containing 0.1 mol L^−1^ KCl for BCPE (curve a) and M-γ-Al_2_O_3_-CPE (curve b). Ac voltage: 5 mV; frequency range: 0.1–10 MHz.

Cyclic voltammetry (CV) and electrochemical impedance spectroscopy (EIS) measurements of M-γ-Al_2_O_3_-CPE were performed in the solution of potassium ferricyanide (Fig. 5). As seen in Fig. 5A, the separation of redox peak potentials (Δ*E*_p_) at M-γ-Al_2_O_3_-CPE get smaller compared with BCPE. The current response of redox peak also increase greatly relative to the BCPE. These phenomena indicate that the modified M-γ-Al_2_O_3_ can not only enlarge the specific surface area of the CPE but also promote the electron transfer. The charge transfer resistance (*R*_ct_) of M-γ-Al_2_O_3_-CPE (Fig. 5B) is quite smaller than that of EIS for BCPE. This result is consistent with the result of CV analysis.

### 3.5. The voltammetric characteristics of Pb^2+^ and Cd^2+^ ion on M-γ-Al_2_O_3_-CPE

**Fig. 6:**
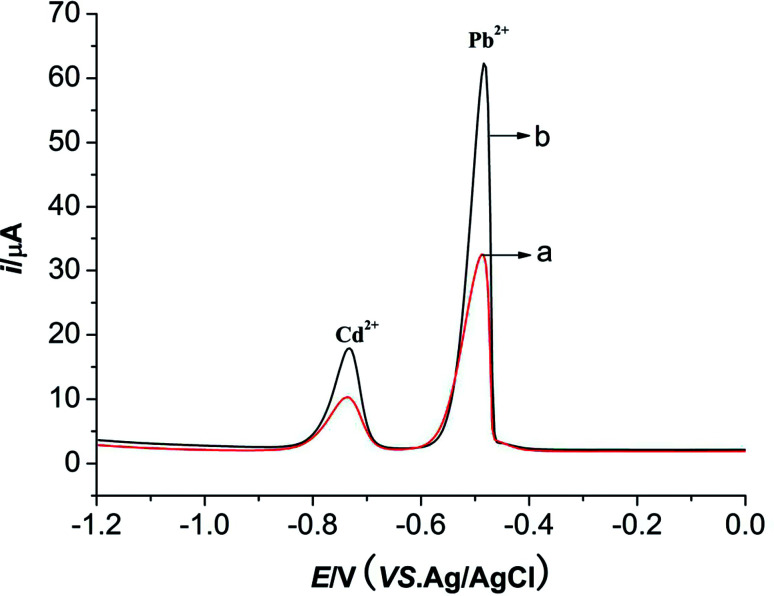
DPVs of Pb^2+^ and Cd^2+^ for BCPE (curve a) and M-γ-Al_2_O_3_-CPE (curve b). Electrochemical conditions are the same as Fig. 4.

Different pulse voltammetry (DPV) was employed to investigate the electrochemical behaviors of Pb^2+^ (10 μmol L^−1^) and Cd^2+^ (10 μmol L^−1^) on M-γ-Al_2_O_3_-CPE and BCPE. The buffer solution used in this system is HAc–NaAc (pH = 4.5). The deposition potential and the deposition time of this system are −1.0 V and 180 s, respectively. As displayed in Fig. 6, sensitivity oxidation peaks of Pb^2+^ and Cd^2+^ can be observed at −0.5 V and −0.75 V, respectively. The peak current responses of two metal ions enhance significantly in the presence of M-γ-Al_2_O_3_ at carbon paste electrode (curve b) compared with BCPE (curve a) implying that the porous M-γ-Al_2_O_3_ exhibits strong adsorption to two metal ions and meanwhile the large surface areas of M-γ-Al_2_O_3_ can accelerate the electron transfer.

### 3.6. Optimization of detection parameters

#### 3.6.1 The supporting electrolyte and pH

Three kinds of buffer solution namely NaAc–HAc, britton–robinson and Na_2_HPO_4_–NaH_2_PO_4_ were tried to be used as the supporting electrolyte of detection system. The results show that better peak shape, resolution and sensitivity were achieved by using buffer solution of NaAc–HAc than that of britton–robinson and Na_2_HPO_4_–NaH_2_PO_4_. So NaAc–HAc buffer solution was selected as the supporting electrolyte for subsequent experiments.

**Fig. 7:**
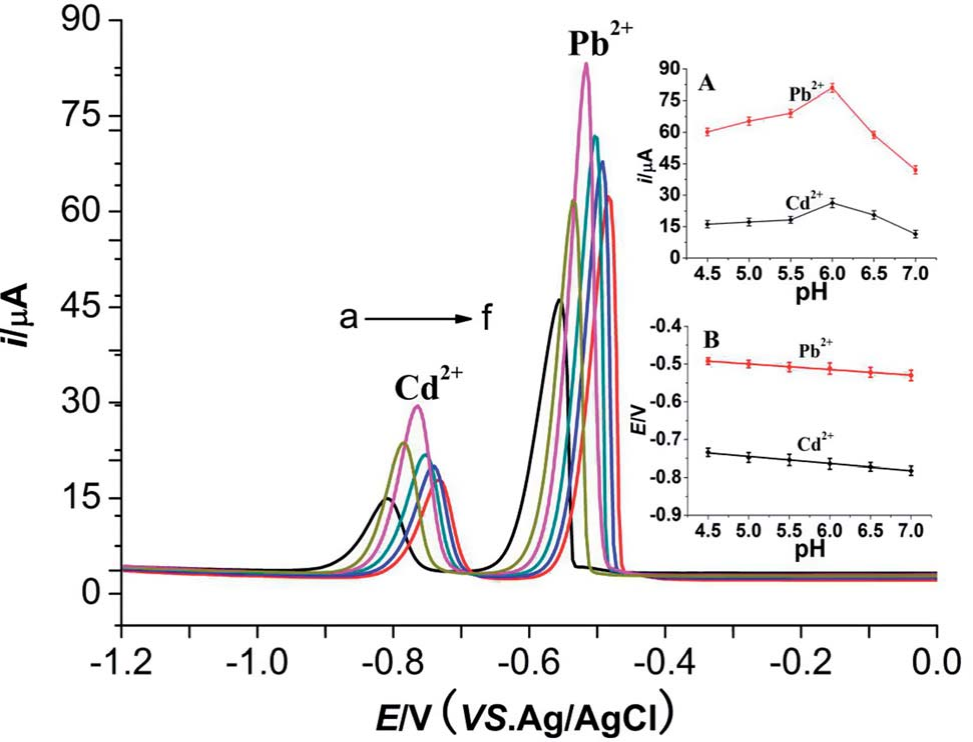
Effect of different pH on 10 μmol L^−1^ Cd^2+^ and 10 μmol L^−1^ Pb^2+^ at a M-Al_2_O_3_ modified carbon paste electrode. Inset (A): plots of *i vs.* pH for Pb^2+^ and Cd^2+^, respectively. Inset (B): plots of *i vs.* pH for Pb^2+^ and Cd^2+^, respectively. pH: a – 7.0, b – 6.5, c – 6.0, d – 5.5, e – 5.0, f – 4.5; other conditions are the same as Fig. 4.

The effects of pH values (NaAc–HAc buffer solution, pH 4.5–7.0) on the peak potential and peak currents of two metal ions are shown in Fig. 7. The peak potentials of two metal ions shift negatively with the increasing of pH values. Good linear relationships between the peak potentials of two metal ions and pH values are obtained. The linear equations are *E*_pa_ (V) = −0.4252 − 0.0149 pH (*R*^2^ = 0.9922) and *E*_pa_ (V) = −0.6532 − 0.0184 pH (*R*^2^ = 0.9914) (inset B), respectively. The peak currents of Pb^2+^ and Cd^2+^ firstly increases and then decreases with the increase of pH values (inset A). They achieve the maximum at pH 6.0. Therefore, pH 6.0 was selected for further study.

#### 3.6.2 The accumulation potential and accumulation time

**Fig. 8:**
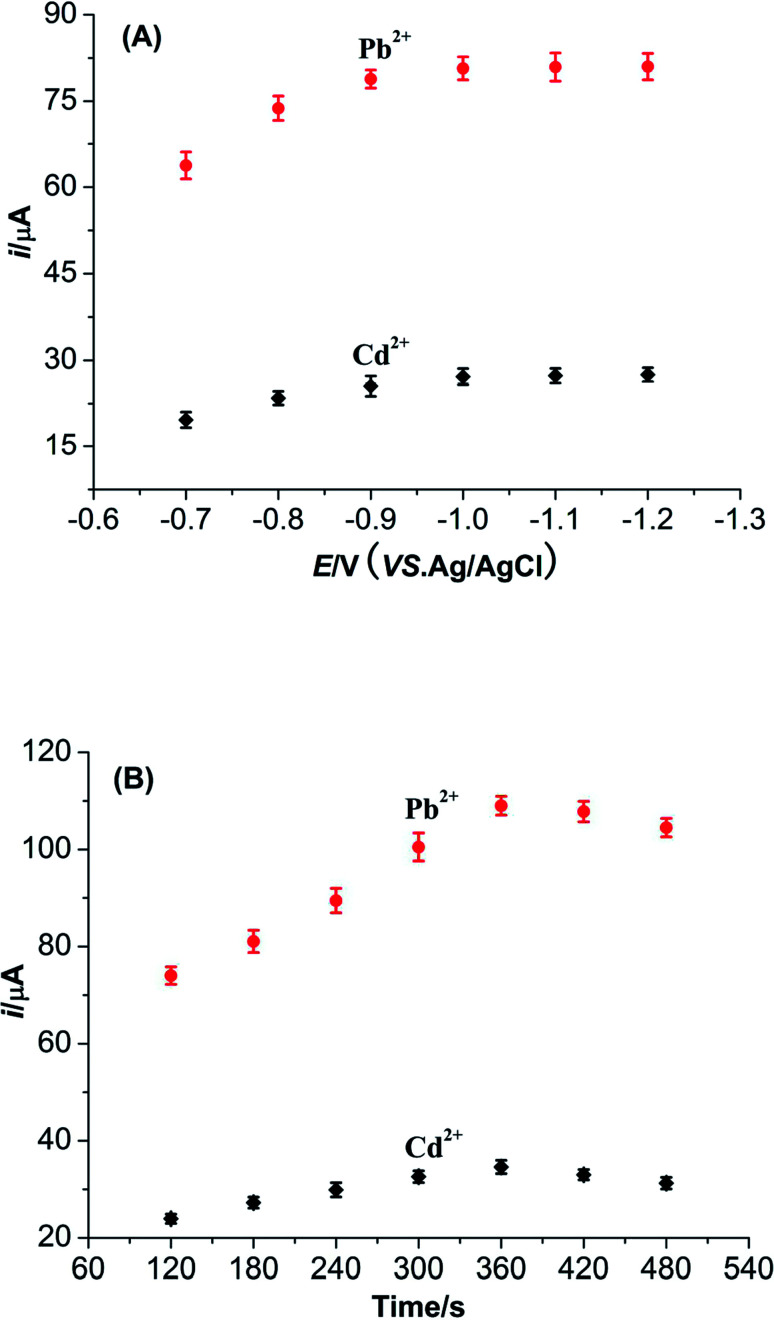
(A) Influence of accumulation potential on the peak currents of Pb^2+^ and Cd^2+^. (B) Influence of accumulation time on the peak currents of Pb^2+^ and Cd^2+^.

The accumulation potential is an important parameter which can affect greatly on the sensitivity of determination for heavy metal ions. Fig. 8 shows the effect of accumulation potential on the peak current of Pb^2+^ and Cd^2+^ with accumulation potential ranging from −0.7 V to −1.2 V. The peak currents of Pb^2+^ and Cd^2+^ can improve obviously with negative shifting of the accumulation potential. When the accumulation potential exceed to −1.0 V, the peak current of two metal ions begin to stabilize. Hence, the accumulation potential is fixed as −1.0 V.

The accumulation time is also another parameter which can apparently influence the detection sensitivities of Pb^2+^ and Cd^2+^. With the increase of accumulation time, the peak currents of Pb^2+^ and Cd^2+^ noticeably increase at first and then decrease gradually after reaching the peak value. In addition, the accumulation time beyond 360 s might result in the distortions of signals for these two metal ions. These results might be explained that the adsorptions of Pb^2+^ and Cd^2+^ on the surface of M-γ-Al_2_O_3_-CPE attain a saturation level. So 360 s is considered to be the accumulation time in this work.

### 3.7. Analytical performances of Pb^2+^ and Cd^2+^

**Fig. 9:**
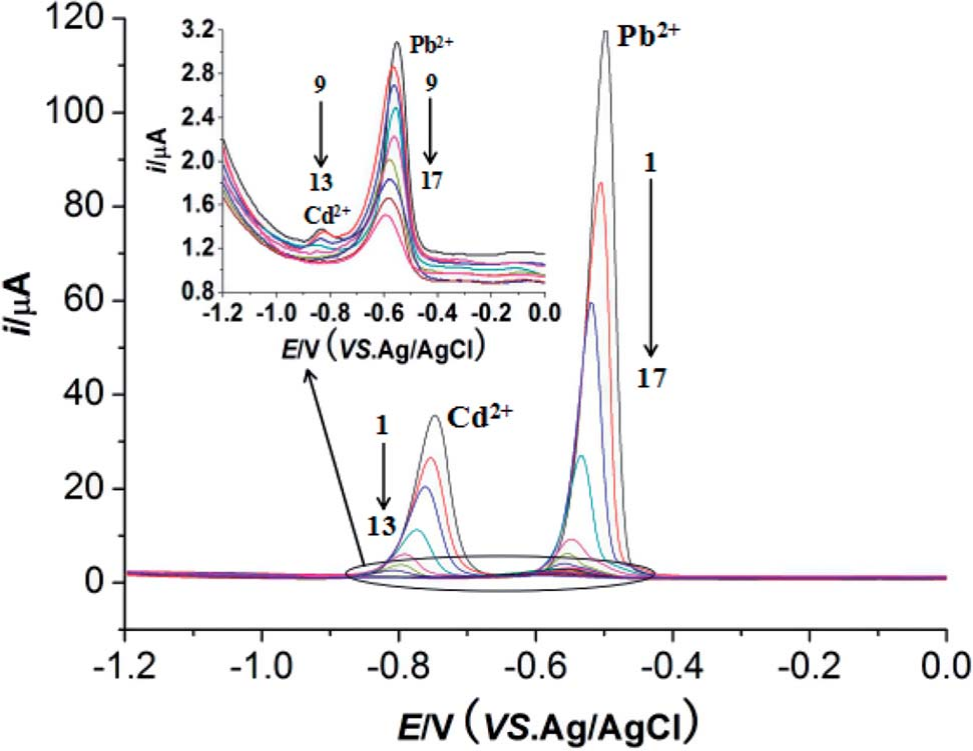
DPVs of of Pb^2+^ and Cd^2+^ with different concentrations at the M-γ-Al_2_O_3_-CPE. Supporting electrolyte: 0.1 mol L^−1^ NaAc–HAc buffer solution (pH 6.0); accumulation time 360 s; concentrations of Pb^2+^ (from 1 → 17): 10, 7.5, 5.0, 2.5, 1.0, 0.75, 0.5, 0.25, 0.1, 0.075, 0.05, 0.025, 0.01, 0.0075, 0.005, 0.0025, 0.001 μmol L^−1^ concentrations of Cd^2+^ (1 → 13): 10, 7.5, 5.0, 2.5, 1.0, 0.75, 0.5, 0.25, 0.1, 0.075, 0.05, 0.025, 0.01 μmol L^−1^. Other conditions are the same as Fig. 4.

**Fig. 10:**
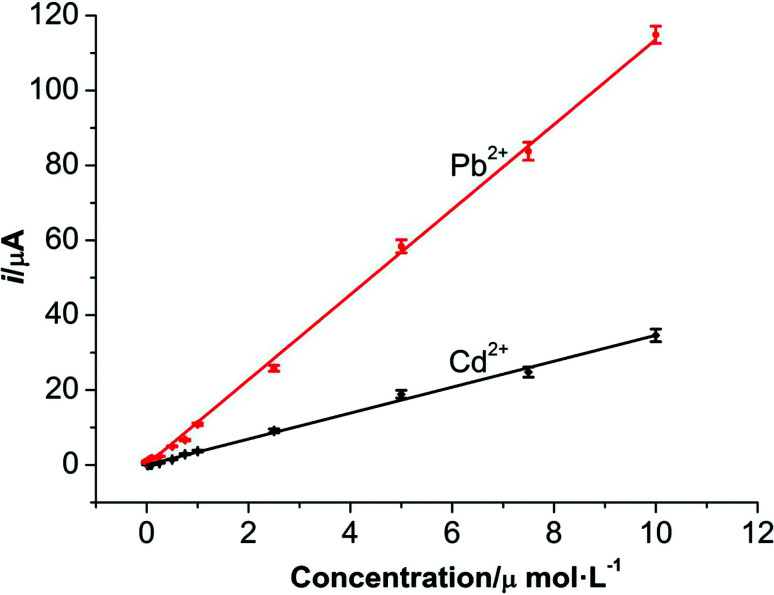
The relationships between the concentrations and current responses of Pb^2+^ and Cd^2+^ at the M-γ-Al_2_O_3_-CPE.

Under the optimum conditions, the determinations of Pb^2+^ and Cd^2+^ were conducted at M-γ-Al_2_O_3_-CPE using DPV method. As described in Fig. 9 and 10, good linear correlation could be found between the peak currents and the concentrations of Pb^2+^ and Cd^2+^. The regression equations, linear response ranges and detection limits are summarized in Table 1. Compared with previous literatures (Table 2), improved or comparable performances have been achieved for the determinations of Pb^2+^ and Cd^2+^ using M-γ-Al_2_O_3_-CPE.

**Table 1:** Analytical parameters for simultaneous determination of Pb^2+^ and Cd^2+^

Analyte	Linear range (μmol L^−1^)	Regression equation *i*_p_^a^, *C*^b^	*R* ^2^	Detection limit (nmol L^−1^)
Pb^2+^	0.001–10	*i*_p_ = 11.349*C* + 0.1034	0.9988	0.20
Cd^2+^	0.01–10	*i*_p_ = 3.4627*C* + 0.0006	0.9971	2.0

a *i*_p_ (μA) is the peak current.

b *C* (μmol L^−1^) is the concentration of the analyte. Electrochemical conditions are the same as Fig. 9.

**Table 2:** Comparison of the response characteristics of different modified electrodes

Electrode materials	Linear range (μmol L^−1^)		Detection limit (nmol L^−1^)		Reference
	Pb^2+^	Cd^2+^	Pb^2+^	Cd^2+^	
Bi-xerogel/GCE^a^	0.0097–0.082	0.0044–0.080	6.3	3.3	32
SNAC/GCE^b^	0.09–5.70	0.09–4.8	5.7	24.0	33
(C–Bi) nanocomposite/CPE^c^	0.0048–0.48	0.0089–0.89	2.9	5.3	34
RGO/Bi/CPE^d^	0.097–0.58	0.18–1.07	2.7	25.0	35
Mo_6_S_9−*x*_I_*x*_ NWs/GCE^e^	0.0072–2.17	0.0045–1.33	2.2	0.9	36
SbNP/MWCNT/CPE^f^	0.048–0.29	0.089–0.53	3.1	6.9	37
CB-15-crown-5/GCE^g^	0.053–0.9	0.14–1.7	16.0	42.0	38
BiOCl/MWCNT/GCE^h^	0.024–0.24	0.044–0.44	2.8	11.0	39
l-cys/GR–CS/GCE^i^	0.05–0.30	0.005–0.60	2.2	1.1	40
BiF_4_/CPE^j^	0.097–0.48	0.18–0.89	5.8	87.0	41
ERGNO/Bi/SPE^k^	0.0048–0.29	0.0089–0.53	3.8	4.5	42
Bi/MGF-Nafion/GCE^l^	0.0024–0.53	0.018–0.62	0.48	4.5	43
Sparked Bi graphite SPE^m^	0.0024–0.058	0.005–0.11	0.97	1.8	44
M-γ-Al_2_O_3_-CPE	0.001–10	0.01–10	0.2	2.0	This work

a Bismuth-dispersed xerogel-based composite films modified glassy carbon electrode.

b Spherical carbon nanoparticle decorated activated carbon modified glassy carbon electrode.

c Bismuth–carbon nanocomposites/carbon paste electrode.

d Reduced graphene oxide/Bismuth/carbon paste electrode.

e Mo_6_S_9−*x*_I_*x*_ nanowires modified glassy carbon electrode.

f Antimony nanoparticle-multiwalled carbon nanotubes composite immobilized at carbon paste electrode.

g 4-Carbox-ybenzo-15-crown-5 modified glassy carbon electrode.

h Bismuth-oxychloride particle-multiwalled carbon nanotube composite modified glassy carbon electrode.

i l-Cysteine/graphene modified glassy carbon electrode.

j Ammonium tetrafluorobismuthate modified carbon paste electrode.

k Electrochemically reduced graphene oxide film modified screen-printed electrode.

l Three-dimensionally interconnected mesoporous graphene framework modified glassy carbon electrode.

m Bi_2_O_3_-modified graphite screen printed electrode.

In order to verify the reproducibility, stability and anti-interference performance of M-γ-Al_2_O_3_-CPE, the measurements were carried out in a NaAc–HAc buffer solution (pH 6.0) containing 1.0 μmol L^−1^ Pb^2+^ and 1.0 μmol L^−1^ Cd^2+^. The reproducibility was evaluated by 8 continuous measurements of peak currents for Pb^2+^ and Cd^2+^. RSDs of the peak currents are 3.5% and 4.3%, respectively. At the same time, five modified carbon paste electrodes were prepared and examined in the same conditions, RSDs of the peak currents are 3.2% and 4.8%, respectively. The M-γ-Al_2_O_3_-CPE was polished by a weighting paper and then eluted by distilled water. The treated electrode was dried and stored in a 4 °C refrigerator. After 7 days, the stripping peak currents for 1.0 μmol L^−1^ Pb^2+^ and 1.0 μmol L^−1^ Cd^2+^ decrease by 6.2% and 7.4%, respectively. These experimental results indicate that the M-γ-Al_2_O_3_-CPE exhibit good reproducibility and stability. Interference experiment was also investigated by adding general coexistence ions to a solution containing 1.0 μmol L^−1^ Pb^2+^ and 1.0 μmol L^−1^ Cd^2+^ in a NaAc–HAc buffer solution (pH 6.0). No evident interference can be found for the determinations of Pb^2+^ and Cd^2+^ in the presence of Na^+^ (1000 μmol L^−1^), K^+^ (1000 μmol L^−1^), Cl^−^ (1000 μmol L^−1^), Br^−^ (1000 μmol L^−1^), F^−^ (1000 μmol L^−1^), Al^3+^ (500 μmol L^−1^), Mg^2+^ (500 μmol L^−1^), Ca^2+^ (500 μmol L^−1^), Zn^2+^ (500 μmol L^−1^) and Mn^2+^ (500 μmol L^−1^) and Fe^3+^ (50 μmol L^−1^) with a maximum allowable error of 5.0%. In addition, Cu^2+^ can interfere with the detection of Cd^2+^, which can compete with Cd^2+^ to reduce on the electrode surface.

### 3.8. Application

To evaluate the application of the proposed M-γ-Al_2_O_3_-CPE, it was used to analyze the concentrations of Pb^2+^ and Cd^2+^ in artificial lake water and tap water samples. The supernatant liquid of artificial lake water and the tap water were collected and diluted 5 times by 0.1 mol L^−1^ NaAc–HAc buffer solution (pH 6.0). The treated water samples were determined by M-γ-Al_2_O_3_-CPE under the optimal experimental conditions. The results are listed in Table 3. The recoveries for these two metal ions are between 97.24% and 103.94%. The water samples were also detected by conventional atomic absorption spectrometry (AAS, WFX-130A, Beijing Rayleigh Analytical Instrument Corp. Ltd.) method. The concentrations of Pb^2+^ and Cd^2+^ were 21.58 ± 0.19 and 8.23 ± 0.11 nmol L^−1^ in tap water, respectively; while those of lake water are 41.67 ± 0.52 and 37.32 ± 0.61 nmol L^−1^, respectively. The measuring results obtained by these two methods are basically the same, indicating a good reliability of the proposed methods.

**Table 3:** Results of the proposed method for the determination of Pb^2+^ and Cd^2+^ in various water samples (*n* = 3)^a^

Sample	Analytes	Added (nmol L^−1^)	Found (nmol L^−1^)	Recovery (%)
Tap water	Pb^2+^	0	21.67 ± 0.15	
		20	40.52 ± 0.66	97.24
	Cd^2+^	0	8.17 ± 0.13	
		50	59.51 ± 0.79	102.3
Lake water	Pb^2+^	0	41.22 ± 0.86	
		20	63.63 ± 1.09	103.94
	Cd^2+^	0	37.01 ± 0.54	
		50	85.72 ± 1.18	98.52

a Electrochemical conditions are the same as Fig. 9.

## 4. Conclusion

In present work, aniline was chosen as an interface protective agent to prepare large surface area M-γ-Al_2_O_3_ which possess a large surface area and a narrow pore-size distribution. The M-γ-Al_2_O_3_ was modified into the carbon paste to fabricate a novel M-γ-Al_2_O_3_-CPE. The proposed electrode has a high sensitivity, stability and reproducibility in the presence of M-γ-Al_2_O_3_. The electrode exhibits excellent performance for the detections of Pb^2+^ and Cd^2+^ in artificial lake water and tap water samples. The developed method will have extensive potential in the field of environmental monitoring.

## Conflicts of interest

There are no conflicts to declare.

## Supplementary Material

## References

[cit1] Kemper T., Sommer S. (2002). Estimate of heavy metal contamination in soils after a mining accident using reflectance spectroscopy. Environ. Sci. Technol..

[cit2] Chang J., Zhou G., Christensen E. R., Heideman R., Chen J. (2014). Graphene-based sensors for detection of heavy metals in water: a review. Anal. Bioanal. Chem..

[cit3] Aragay G., Merkoci A. (2012). Nanomaterials application in electrochemical detection of heavy metals. Electrochim. Acta.

[cit4] Zhang J. Y., Fang J. L., Duan X. C. (2016). Determination of cadmium in water samples by fast pyrolysis–chemical vapor generation atomic fluorescence spectrometry. Spectrochim. Acta.

[cit5] Luo H., Wang X. Y., Dai R., Liu Y., Jiang X., Xiong X. L., Huang K. (2017). Simultaneous determination of arsenic and cadmium by hydride generation atomic fluorescence spectrometry using magnetic zero-valent iron nanoparticles for separation and pre-concentration. Microchem. J..

[cit6] Shaltout A. A., Boman J., Welz B., Castilho I. N. B., Al Ashkar E. A., Gaita S. M. (2014). Method development for the determination of Cd, Cu, Ni and Pb in PM 2.5 particles sampled in industrial and urban areas of Greater Cairo, Egypt, using high-resolution continuum source graphite furnace atomic absorption spectrometry. Microchem. J..

[cit7] Bagheri H., Afkhami A., Saber-Tehrani M., Khoshsafar H. (2012). Preparation and characterization of magnetic nanocomposite of Schiff base/silica/magnetite as a preconcentration phase for the trace determination of heavy metal ions in water, food and biological samples using atomic absorption spectrometry. Talanta.

[cit8] Lin M. L., Jiang S. J. (2013). Determination of As, Cd, Hg and Pb in herbs using slurry sampling electrothermal vaporisation inductively coupled plasma mass spectrometry. Food Chem..

[cit9] Lamsal R. P., Beauchemin D. (2015). Estimation of the bio-accessible fraction of Cr, As, Cd and Pb in locally available bread using on-line continuous leaching method coupled to inductively coupled plasma mass spectrometry. Anal. Chim. Acta.

[cit10] Zhou Q. X., Lei M., Liu Y. L., Wu Y. L., Yuan Y. Y. (2017). Simultaneous determination of cadmium, lead and mercury ions at trace level by magnetic solid phase extraction with Fe@Ag@dimercaptobenzene coupled to high performance liquid chromatography. Talanta.

[cit11] Schumacher P. D., Fitzgerald K. A., Schenk J. O., Clark S. B. (2011). Preconcentration off-elements from aqueous solution utilizing a modified carbon paste electrode. Anal. Chem..

[cit12] Svancara I., Vytras K., Kalcher K., Walcarius A., Wang J. (2009). Carbon paste electrodes in facts, numbers, and notes: a review on the occasion of the 50-years jubilee of carbon paste in electrochemistry and electroanalysis. Electroanalysis.

[cit13] Afkhami A., Madrakian T., Ghaedi H., Khanmohammadi H. (2012). Construction of a chemically modified electrode for the selective determination of nitrite and nitrate ions based on a new nanocomposite. Electrochim. Acta.

[cit14] Yan B. B., Li W. Q., Tao J. Y., Xu N. G., Li X. P., Chen G. Y. (2017). Hydrogen production by aqueous phase reforming of phenol over Ni/ZSM-5 catalysts. Int. J. Hydrogen Energy.

[cit15] Raoof J. B., Chekin F., Ehsani V. (2015). Palladium-modified mesoporous silica SBA-15 modified in carbon-paste electrode as a sensitive voltammetric sensor for detection of oxalic acid. Sens. Actuators, B.

[cit16] Oord R., Weckhuysen B. M. (2016). Chapter 10 –Cu-Zeolite Selective Catalytic Reduction Catalysts for NO_*x*_ Conversion. Zeolites Zeolite-Like Mater..

[cit17] Bardakci B., Bahceli S. (2013). Adsorption of *n*-propyl mercaptan on Pb/LTA zeolites. Prot. Met. Phys. Chem. Surf..

[cit18] Sun J. Y., Gan T., Deng Y. P., Shi Z. X., Lv Z. (2015). Pt nanoparticles-functionalized hierarchically porous Al_2_O_3_ hollow spheres based electrochemical sensor for ultrasensitive guaiacol detection. Sens. Actuators, B.

[cit19] Jayaraman V., Prabhu E., Sree Rama Murthy A., Clinsha P. C., Gnanasekar K. I., Gnanasekaran T. (2014). Na-β-Al_2_O_3_ based sensor for sodium aerosol. Sens. Actuators, B.

[cit20] Yu J. J., Ma J. R., Zhao F. Q., Zeng B. Z. (2007). Direct electron-transfer and electrochemical catalysis of hemoglobin immobilized on mesoporous Al_2_O_3_. Electrochim. Acta.

[cit21] Martena K. L., Grant S. M., Jaroniec M. (2012). Poly(ethylene oxide)–poly(butylene oxide)–poly(ethylene oxide)-templated synthesis of mesoporous alumina: effect of triblock copolymer and acid concentration. ACS Appl. Mater. Interfaces.

[cit22] Mitra A., Jana D., De G. (2012). Facile synthesis of hexagonally ordered mesoporous aluminum oxide thin films with high catalytic activity. Microporous Mesoporous Mater..

[cit23] Grant S. M., Jaroniec M. (2012). Effect of acid concentration on pore size in polymer-templated mesoporous alumina. J. Mater. Chem..

[cit24] Zheng X. L., Sun Q. P., Liu F., Zheng Y., Weng J. B. (2014). Effect of p-aminobenzoic on synthesizing ordered mesoporous alumina *via* the sol-gel method. J. Porous Mater..

[cit25] Huang F., Zheng Y., Cai G. H., Zheng Y., Xiao Y. H., Wei K. M. (2010). A new synthetic procedure for ordered mesoporous-alumina with a large surface area. Scr. Mater..

[cit26] Pan F., Lu X. C., Wang T., Wang Y., Zhang Z. M., Yan Y., Yang S. P. (2013). Synthesis of large-mesoporous Al_2_O_3_ from coal-series kaolin at room temperature. Mater. Lett..

[cit27] Yuan Q., Yin A. X., Luo C., Sun L. D., Zhang Y. W., Duan W. T., Liu H. C., Yan C. H. (2008). Facile synthesis for ordered mesoporous γ-aluminas with high thermal stability. J. Am. Chem. Soc..

[cit28] Wu Q. L., Zhang F., Yang J. P., Li Q., Tu B., Zhao D. Y. (2011). Synthesis of ordered mesoporous alumina with large pore sizes and hierarchical structure. Microporous Mesoporous Mater..

[cit29] Sun Q., Zheng Y., Li Z. H., Zheng Y., Xiao Y. H., Cai G. H. (2012). Synthesis of ordered mesoporous γ-alumina influenced by the interfacial protector. Mater. Lett..

[cit30] Sun Q., Zheng Y., Li Z. H., Zheng Y., Xiao Y. H., Cai G. H., Wei K. M. (2013). Studies on the improved thermal stability for doped ordered mesoporous γ-alumina. Phys. Chem. Chem. Phys..

[cit31] Yuan Q., Duan H. H., Li L. L., Li Z. X., Duan W. T., Zhang L. S., Song W. G., Yan C. H. (2010). Homogeneously dispersed ceria nanocatalyst stabilized with ordered mesoporous alumina. Adv. Mater..

[cit32] Dimovasilis P. A., Prodromidis M. I. (2013). Bismuth-dispersed xerogel-based composite films for trace Pb(II) and Cd(II) voltammetric determination. Anal. Chim. Acta.

[cit33] Madhu R., Sankar K. V., Chen S. M., Selvan R. K. (2014). Eco-friendly synthesis of activated carbon from dead mango leaves for the ultrahigh sensitive detection of toxic heavy metal ions and energy storage applications. RSC Adv..

[cit34] Gich M., Fernandez-Sanchez C., Cotet L. C., Niu P., Roig A. (2013). Facile synthesis of porous bismuth-carbon nanocomposites for the sensitive detection of heavy metals. J. Mater. Chem. A.

[cit35] Sahoo P. K., Panigrahy B., Sahoo S., Satpati A. K., Li D., Bahadur D. (2013). In situ synthesis and properties of reduced graphene oxide/Bi nanocomposites: as an electroactive material for analysis of heavy metals. Biosens. Bioelectron..

[cit36] Lin H., Li M., Mihailovič D. (2015). Simultaneous determination of copper, lead, and cadmium ions at a Mo_6_S_9−*x*_I_*x*_ nanowires modified glassy carbon electrode using differential pulse anodic stripping voltammetry. Electrochim. Acta.

[cit37] Ashrafi A. M., Cerovac S., Mudrić S., Guzsvány V., Husáková L., Urbanová I., Vytřas K. (2014). Antimony nanoparticle-multiwalled carbon nanotubes composite immobilized at carbon paste electrode for determination of trace heavy metals. Sens. Actuators, B.

[cit38] Serrano N., González-Calabuig A., del Valle M. (2015). Crown ether-modified electrodes for the simultaneous stripping voltammetric determination of Cd(II), Pb(II) and Cu(II). Talanta.

[cit39] Cerovac S., Guzsvány V., Kónya Z. (2015). Trace level voltammetric determination of lead and cadmium in sediment pore water by a bismuth-oxychloride particle-multiwalled carbon nanotube composite modified glassy carbon electrode. Talanta.

[cit40] Zhou W., Li C., Sun C., Yang X. (2016). Simultaneously determination of trace Cd^2+^ and Pb^2+^ based on l-cysteine/graphene modified glassy carbon electrode. Food Chem..

[cit41] Sopha H., Baldrianová L., Tesařová E. (2010). A New Type of Bismuth Electrode for Electrochemical Stripping Analysis Based on the Ammonium Tetrafluorobismuthate Bulk-Modified Carbon Paste. Electroanalysis.

[cit42] Ping J., Wang Y., Wu J., Ying Y. (2014). Development of an electrochemically reduced graphene oxide modified disposable bismuth film electrode and its application for stripping analysis of heavy metals in milk. Food Chem..

[cit43] Xiao L. L., Wang B. W., Ji L., Yuan Q. H., Hu G. Z., Dong A. G., Gan W. (2016). An efficient electrochemical sensor based on three-dimensionally interconnected mesoporous graphene framework for simultaneous determination of Cd(II) and Pb(II). Electrochim. Acta.

[cit44] Riman D., Jirovsky D., Hrbac J., Prodromidis M. I. (2015). Green and facile electrode modification by spark discharge: bismuth oxide-screen printed electrodes for the screening of ultra-trace Cd(II) and Pb(II). Electrochem. Commun..

